# Diagnostic value of copeptin combined with hypersensitive cardiac troponin T detection in early acute myocardial infarction: A protocol of randomized double-blind diagnostic trial

**DOI:** 10.1097/MD.0000000000023949

**Published:** 2021-01-08

**Authors:** Yan Yang, Songtao Gao, Qiuju Fang, Jing Yang

**Affiliations:** Department of Cardiovascular Medicine, Heilongjiang Provincial Hospital, Harbin, Heilongjiang Province, China.

**Keywords:** acute myocardial infarction, copeptin, Hs-cTnT, protocol, RCT

## Abstract

**Background:**

The sensitivity and specificity of the routine detection of acute myocardial infarction (AMI) in early diagnosis are not high, which can not meet the clinical needs. Copeptin combined with hypersensitive cardiac troponin T (hs-cTnT) is a new detection scheme, and its value in the early diagnosis of acute myocardial infarction is still unclear. Accordingly, the aim of this study is to evaluate the diagnostic value of copeptin combined with hypersensitive troponin T detection in early acute myocardial infarction.

**Methods:**

This is a prospective, randomized; double-blind diagnostic trial to investigate the diagnostic value of copeptin combined with hypersensitive troponin T detection in early acute myocardial infarction. Approved by the clinical research ethics of our hospital. Patients were randomly divided into one of 2 test protocols: (A) copeptin combined with hs-cTnT group and (B) cardiac troponin I (cTnI) group. Patients, doctors, nurses, inspectors, and data-gathering assistants were blinded to group allocation. We will focus on the sensitivity comparison of the 2 detection methods at different time periods and the sensitivity and specificity comparison of the two detection methods. Data were analyzed using the statistical software package SPSS version 25.0 (Chicago, IL).

**Discussion:**

The purpose of this study is to evaluate the diagnostic value of copeptin combined with hypersensitive cardiac troponin T detection in early acute myocardial infarction. The results of this study will establish clinical evidence for the detection of high sensitivity cardiac troponin T in the early diagnosis of acute myocardial infarction.

**Ethics and dissemination:**

Private information from individuals will not be published. This systematic review also does not involve endangering participant rights. Ethical approval was not required. The results may be published in a peer-reviewed journal or disseminated at relevant conferences.

**OSF Registration number:**

DOI 10.17605/OSF.IO/6TE5Z.

## 1 Introduction

Acute myocardial infarction (AMI) is an acute coronary syndrome in which blocked coronary artery causes insufficient blood supply to the heart muscle and impairs heart function, has the characteristics of rapid progression and high mortality,^[1]^ is the leading cause of cardiovascular death worldwide.^[2]^ Rapid and accurate early diagnosis of AMI is the key to reduce mortality and improve prognosis of patients.^[3]^

In the previous clinical diagnosis, AMI was mainly diagnosed based on medical history, physical examination, electrocardiogram, echocardiography and other examinations, but all of these examinations had certain defects and their accuracy was not high, while myocardial necrosis markers such as C reactive protein (CRP), myoglobin (Mb), creatine kinase isoenzyme (CK-MB) have poor sensitivity and specificity in the early stage of the disease.^[4]^ Although cardiac troponin (cTn) has become a recognized indicator for the diagnosis of AMI, the blood concentration reflecting myocardial necrosis cannot be detected within 1 hour after the onset of AMI symptoms, and the level of troponin begins to rise 3 to 6 hours after the occurrence of myocardial injury, appearing the “troponin blindness stage”,^[5]^ since cTn lacks sensitivity in the early stages of myocardial injury, it is particularly important to add a marker for rapid diagnostic AMI. Copeptin is a newly discovered marker of myocardial necrosis. Clinical studies have confirmed that copeptin begin to rise in early AMI, earlier than troponin,^[6]^ breaking the “troponin blindness stage” and making up for defects in early atypical changes in electrocardiogram. Early diagnosis and prognosis evaluation of AMI can be carried out quickly.

Some studies have pointed out that although the level of copeptin in AMI patients is significantly increased, because of the lack of specificity, it should not be considered as a single biomarker for the diagnosis of suspected AMI patients.^[7]^ The sensitivity of hypersensitive cardiac troponin (hs-cTn) is higher than that of traditional troponin. At present, the diagnostic value of hs-cTn combined with copeptin in early detection of AMI is not clear. The aim of this study is to investigate the early diagnostic value of hs-cTnT combined with copeptin in early detection of AMI.

## 2 Materials and methods

### 2.1 Study design

**Figure 1:**
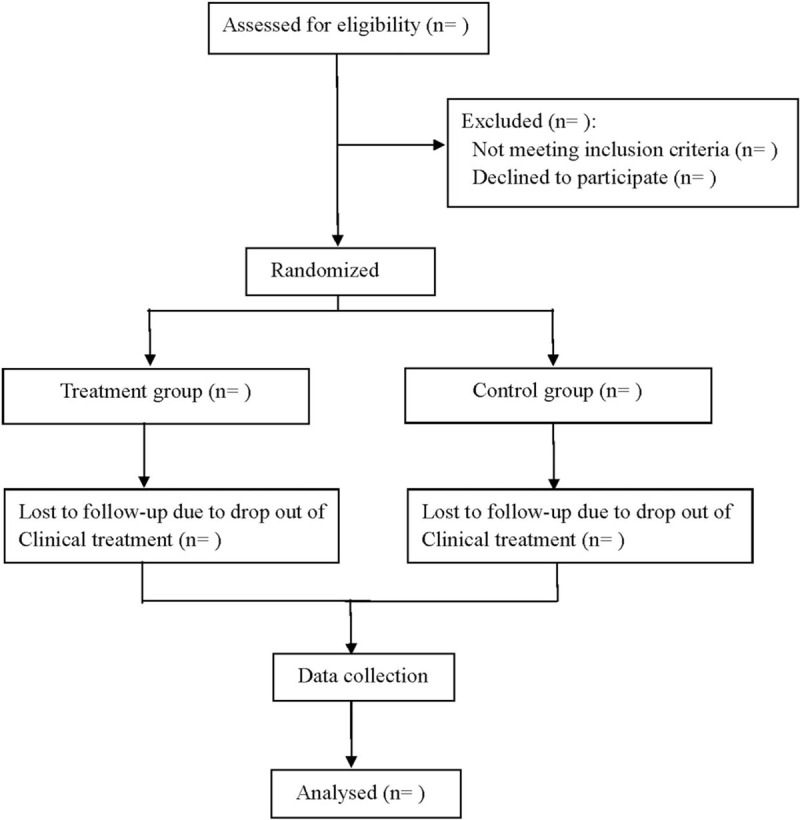
Flow diagram.

This is a prospective randomized double-blind diagnostic trial to study the accuracy of copeptin combined with hs-cTnT in the diagnosis of early acute myocardial infarction. This study follows the guidelines of Standards for Reporting of Diagnostic Accuracy Studies,^[8]^ And we followed the Consolidated Standards of Reporting Trials (CONSORT) guidelines for reporting randomized trials and provided a CONSORT flow diagram (Fig. 1).

### 2.2 Ethics and registration

This protocol is in accordance with Helsinki Declaration and approved by the Clinical Research Ethics Committee of our hospital. This experiment has been registered in open science framework (OSF) (Registration number: DOI 10.17605/OSF.IO/6TE5Z). Prior to the trial, written informed consent will be obtained from all patients. Personal information about potential participants and registered participants will be collected, Shared, and kept in a separate repository to protect confidentiality before, during, and after the trial.

### 2.3 Sample size

Estimation of sample contents for both test and control groups will be obtained based on anticipated sensitivity according to Buderer formula:^[9,10]^

$$n=\frac{Z_{1-\frac{a}{2}}^{2}\times S_{N}\times \left(1-S_{N}\right)}{L^{2}\times Prevalence}$$

Where n = required sample size; S_N_ = anticipated sensitivity; *α* = size of the critical region (1 – α is the confidence level); Z_1-α/2_ = standard normal deviate corresponding to the specified size of the critical region (α), and L = absolute precision desired on either side (half-width of the confidence interval) of sensitivity or specificity. According to the sensitivity of the test group to be 90% and that of the control group to be 75%, the sensitivity of the control group to be 0.8, α=0.05, Z_1-α/2_ = 1.96, L = 0.1, it is calculated that at least 43 persons are required to be included on the test group and 90 persons on the control group. At last, 90 persons are selected from the test group and the control group respectively.

### 2.4 Patients

Inclusion criteria: Clinical examination meets the diagnostic criteria of AMI^[11]^; Regardless of gender, age ≥18; Be to the hospital within 2 hours after onset of chest pain; Be all received AMI therapy for the first time and had no old myocardial infarction; Voluntary participants with informed consent of patients and their families.

Exclusion criteria:

1. Patients with severe arrhythmia;
2. Patients with congenital heart disease and heart valve organic lesions;
3. Patients with malignant tumors examined;
4. Patients with heart, liver and kidney dysfunction;
5. Unable to understand the research programme or unwilling participants.

### 2.5 Randomization and blinding

Patients were randomly divided into one of 2 test protocols:

1. Copeptin combined with hs-cTnT group,
2. cTnI group.

Randomization was performed without any stratification. Randomization listings were prepared with a probability of 1:1 and after that, randomized numbers were printed according to the results of the randomization. After the patient had given consent, the members of the inpatient clinical research center divided the patients into 2 groups according to the parity of the assigned numbers, the odd number was the test group, and the even number was the control group. Patients, doctors, inspectors, nurses and research assistants collecting data were blinded to group allocation.

### 2.6 Methods

1. After admission, 5 ml cubital venous blood was collected from all patients at 0 hours, 4 hours and 6 hours, respectively. According to the digital markers assigned at admission, the blood was centrifuged at 3000 r/minutes for 10 minutes, and the plasma was collected and stored at -70°C for testing.
2. cTnI detection: Immunofluorescence assay was used, the kit was produced by Roche in Germany, cTnI value <0.05ng/ml was used as the diagnostic cut-off point; (2) hs-cTnT detection: Electrochemical luminescence method was used on roche Cobas 601 automatic chemiluminescence instrument. The kit was Roche fifth generation trotropoin reagent, and hs-cTnT value ≤14 ng/L as the diagnostic cut-off point^[12]^; (3) copeptin detection: Using enzyme linked immunosorbent assay (ELISA), the kit was produced by phoenix pharmaceuticals in the United States, and the copeptin value <14 pmol/L as the diagnostic cut-off point.^[6,13]^
3. All patients underwent coronary angiography as the gold standard for diagnostic validation: CAG was performed by an experienced interventional cardiologist using the standard Judkins method, with at least 3 multi-position exposures per vessel.

### 2.7 Data collection and management

All data will be collected separately by 2 assistants, and all data will be stored and kept separately. The access to the database will be restricted to the researchers in this study team.

### 2.8 Statistical analysis

SPSS 25.0 was used for statistical analysis of the data and normality test of the measurement data. The normal distribution data were expressed as $\bar{x}\pm S$, and the comparison between groups was performed by one-way ANOVA. Non-normal distribution data were represented by median and quartile, rank sum test was used for comparison between groups, Chi-Squared test was used for comparison between count data sets. Receiver operating characteristic curve (ROC) was used to screen the best diagnostic critical point value, and its sensitivity and specificity were calculated to evaluate the diagnostic effect. The area under the 2 ROC curves (AUC) was compared by *Z* test. *P* < .05 was statistically significant.

## 3 Discussion

Since chest pain and other symptoms account for about 5% to 10% of the total number of emergency patients,^[14]^ accurate diagnosis of AMI in chest pain patients in the early stage is of great clinical value in improving the prognosis of patients and reducing their mortality.

Troponin T is a regulatory protein for myocardial tissue contraction and a biochemical marker of myocardial injury and necrosis. It is usually detected from the blood 4 to 6 hours after chest pain occurs. Traditional cTnT detection methods have lower sensitivity and affect the early diagnosis of AMI, while hs-cTnT have higher sensitivity than traditional detection methods.^[15]^ Hs-cTnT has high sensitivity to myocardial injury and strong specificity, can appear in the early stage of AMI, and can exist in the blood stream for a long period of time. It is the most favorable marker for early diagnosis and prognosis evaluation of AMI.^[16]^ The hypersensitive troponin will rise abnormally when there is damage of cardiomyocytes and the sensitivity is high, serum level of hypersensitive troponin T is positively correlated with the degree of myocardial injury in acute myocardial infarction.^[17]^

Copeptin, a part of the original C-terminal of arginine vasopressin (AVP), is positively correlated with AVP in the normal population and patients with cardiopulmonary and vascular diseases, and is an acute endogenous stress marker. Its stability in vivo is stronger than AVP, and it is easy to measure. Therefore, copeptin can replace AVP as a clinical early-warning marker of cardiovascular disease.^[18]^ The plasma copeptin level peaks immediately after AMI, and decreases gradually within 6 hours, with the largest change range within 0 to 3 hours.^[19]^ However, the copeptin specificity is low, and it can increase in acute or chronic stress state, such as respiratory tract infection, chronic obstructive pulmonary disease, heart failure, critical illness and so on, which has certain influence on the early diagnosis of AMI.^[20]^ The combination of copeptin and hs-cTnT detection is expected to improve specificity and accuracy in early diagnosis of acute myocardial infarction.

Cardiac troponin I remains the preferred cardiac marker for AMI diagnosis, according to guidelines published by the European Society of Cardiology (ESC), the American College of Cardiology (ACC), and the American Heart Association (AHA).^[21,22]^ Therefore, we used cTnI as a control group to evaluate the clinical value of copeptin combined hs-cTnT in the diagnosis of early acute myocardial infarction.

This study may have the following limitations: because this is a single central regional experimental study, the inclusion of the population may be relatively single, there is a certain bias; factors such as age may have a certain impact on the results.

## Author contributions

**Data collection:** Yan Yang and Qiuju Fang

**Literature retrieval:** Songtao Gao

**Supervision:** Songtao Gao and Qiuju Fang

**Software operating:** Qiuju Fang

**Funding support:** Jing Yang

**Writing – original draft:** Yan Yang and Songtao Gao

**Writing – review & editing:** Yan Yang and Jing Yang
